# Integrating Dentistry into Interprofessional Healthcare: A Scoping Review on Advancing Collaborative Practice and Patient Outcomes

**DOI:** 10.3390/healthcare13212780

**Published:** 2025-11-01

**Authors:** Man Hung, Wendy C. Birmingham, Madeleine Tucker, Connor Schwartz, Amir Mohajeri

**Affiliations:** 1 College of Dental Medicine, Roseman University of Health Sciences, South Jordan, UT 84095, USA; 2 David Eccles School of Business, University of Utah, Salt Lake City, UT 84112, USA; 3 Division of Public Health, University of Utah, Salt Lake City, UT 84108, USA; 4 Department of Psychology, Brigham Young University, Provo, UT 84602, USA; 5 Library, Roseman University of Health Science, South Jordan, UT 84095, USA; 6 Library, Noorda College of Osteopathic Medicine, Provo, UT 84606, USA

**Keywords:** interprofessional healthcare, patient-centered care, dentistry, collaboration, integration

## Abstract

**Background**: Interprofessional collaboration is vital for comprehensive, patient-centered care. Despite growing recognition of oral–systemic health links, the integration of dentists into healthcare teams remains limited. This scoping review mapped existing evidence on dental professionals’ roles within interprofessional healthcare, identifying key benefits, barriers, and facilitators. **Methods**: A systematic search of PubMed, SCOPUS, and Web of Science identified English-language studies (2014 to 2024) focused on collaboration between dental and non-dental providers. Studies addressing oral–systemic health without team-based integration were excluded. Screening and data charting followed the PRISMA-ScR framework using JBI data extraction and critical appraisal tools. Data were synthesized thematically by collaboration model, outcomes, and influencing factors. **Results**: Nine studies met the inclusion criteria. Integrating dental professionals into healthcare teams improved patient outcomes, quality of life, and satisfaction. Effective models included nurse practitioner–dentist partnerships and medical–dental collaboration in pediatrics and chronic disease care. Barriers included poor communication, lack of interoperable electronic health records, role ambiguity, and limited interprofessional training. Key facilitators were supportive policies, integrated care structures, professional education, and strong team communication. **Conclusions**: Integrating dentists into interprofessional teams enhances healthcare delivery and patient outcomes. However, significant barriers remain. Addressing communication gaps, implementing shared health records, and expanding interprofessional education are essential steps toward more cohesive care. Future research should evaluate scalable integration frameworks and incorporate patient perspectives to inform team-based care.

## 1. Introduction

The World Health Organization defines interprofessional collaboration (IPC) as the process by which multiple healthcare professionals work together with patients, families, caregivers, and communities to deliver coordinated, patient-centered care [1]. Health professionals involved include mental health providers, specialty and primary care physicians, nurse practitioners, registered dietitians, social workers, psychologists, and pharmacists [2]. Dentists are essential members of this network, contributing to disease prevention, early detection, and health promotion throughout the lifespan. The FDI World Dental Federation emphasizes oral health as integral to overall health, defining it as the capacity to speak, smile, chew, and swallow effectively [3]. Poor oral health, particularly periodontal disease and chronic inflammation, has been linked to systemic conditions such as cardiovascular disease, diabetes, respiratory illness, and cognitive decline [4,5], underscoring the need to integrate dental professionals into broader healthcare systems.

Opportunities for integration have been documented across populations and chronic conditions. Primary care providers frequently interact with at-risk groups such as children, pregnant individuals, and patients with chronic diseases like diabetes [6]. Research shows that children are more likely to attend dental appointments when referred by a primary care provider [7,8], and pediatric patients tend to see their medical providers more regularly than their dentists [9]. National survey data confirm these patterns: younger children have more frequent physician visits, while older children are more likely to see a dentist, suggesting an opportunity for both professions to reinforce each other’s preventive roles [10]. The increased medical contact correlates with a lower incidence of dental decay among children [11]. Interprofessional interventions in pediatric and maternal–child health settings also demonstrate measurable benefits. For example, a randomized trial in Peru found that collaboration between nurses and dentists within mother–child health clinics significantly reduced early childhood caries [12]. Additionally, it is now common for pediatric providers to apply fluoride varnish during routine checkups, a preventive practice that strengthens teeth and reduces decay [13]. Similarly, collaborative efforts in underserved communities also demonstrate feasibility and effectiveness in improving oral health outcomes [14].

Among pregnant individuals, misconceptions about dental care safety [15] limit care use despite a higher risk for gingivitis and caries [16,17]. Obstetricians and gynecologists, as well as other maternal health professionals, can play a key role in addressing these gaps through coordinated patient education [18]. Similarly, people with diabetes, who are more prone to periodontal disease, often underutilize preventive dental services and engage less in recommended oral hygiene practices [19,20]. Because diabetic patients are typically managed by multidisciplinary teams—including physicians, endocrinologists, dietitians, podiatrists, diabetes educators, and optometrists—each professional can reinforce oral health promotion [21]. Collectively, these examples show how IPC can close preventive and therapeutic gaps in oral–systemic health.

Beyond medical settings, community-based and social service professionals can play a role in oral health promotion. Older adults benefit when nonmedical service providers, such as home care aides or community nutrition programs, incorporate oral health assessments or referral prompts into their workflows [22]. Such intersectoral collaboration demonstrates that diverse professional networks can advance oral health more effectively than siloed care models [23]. Community–academic partnerships and extramural dental education rotations further expand access to care and foster IPC in underserved areas [24].

Dentists are also vital in detecting systemic conditions manifesting in the oral cavity. The U.S. Surgeon General’s report emphasizes their unique role in early disease identification [25]. For instance, dentists can detect signs of eating disorders and refer patients for medical and mental health evaluation [26]. Oral indicators such as halitosis, bleeding gums, or chronic inflammation may suggest diabetes [27]; bone loss in the jaw may indicate osteoporosis [28]; pallor of oral tissues or persistent ulcers may suggest anemia or nutritional deficiencies [29]; and recurrent oral yeast infections could indicate HIV [30]. Emerging evidence also highlights their role in identifying systemic complications of neurological diseases such as Parkinson’s, where oral dysfunction can accelerate overall decline [31]. These findings underscore the importance of integrating dental and medical providers through IPC.

Dentists also contribute to emergency and public health responses, participating in disaster teams and supporting immunization, triage, and preparedness efforts [32]. Effective IPC depends on trust, psychological safety, and shared mental models within healthcare teams [33]. Despite these benefits, dental–medical integration remains limited [13,34]. Barriers include insufficient resources, unclear role definitions, inadequate training, policy gaps, poor interdisciplinary education, and implementation challenges [35]. Time constraints during medical appointments [34] and the inability to share integrated electronic health records (i.e., the interoperable systems that allow medical and dental providers to access and exchange patient data in real time) further hinder collaboration [36].

Prior reviews offer insights into oral–systemic health and interprofessional education (IPE) but rarely examine how dentists are embedded within healthcare delivery teams. Rawlinson et al. [2] identify general IPC barriers (e.g., communication, role clarity, culture) but do not focus on dentistry. Khabeer et al. [37] and Alqutaibi et al. [38] examine IPE outcomes in dental education but not clinical practice. Thus, existing reviews stop short of analyzing operational models of interprofessional care involving dentists.

This review addressed that gap by examining the models, benefits, barriers, and facilitators of dentist integration into interprofessional healthcare teams in U.S. settings. This U.S. focus allowed a targeted examination of healthcare delivery systems shaped by similar policy, reimbursement, and technological infrastructures.

## 2. Methods

This scoping review was preregistered on the Open Science Framework (OSF; https://osf.io/76uzj, accessed on 9 September 2025) and utilized articles sourced from PubMed, SCOPUS, and Web of Science—databases selected for their extensive and relevant collections of biomedical research on interprofessional collaboration. The scoping review approach was chosen because the evidence base on dental integration within interprofessional healthcare remains conceptually broad and methodologically diverse, spanning multiple study designs, populations, and care models. This approach allows for mapping the range, nature, and characteristics of existing research rather than evaluating intervention effectiveness, which aligns with the study’s exploratory objectives. Article selection followed the Preferred Reporting Items for Systematic Reviews and Meta-Analyses Extension for Scoping Review (PRISMA-ScR) [39] guidelines, ensuring transparency and reproducibility. To assess methodological quality, we applied the Joanna Briggs Institute Critical Appraisal Tool [40] for Scoping Reviews, which evaluates key domains such as study design appropriateness, data clarity, analytical rigor, and alignment between objectives and conclusions.

As detailed in Table 1, inclusion and exclusion criteria were established before data extraction. Inclusion criteria required studies to (1) be peer-reviewed; (2) focus on interprofessional healthcare teams involving dentists; (3) report benefits, challenges, or outcomes related to such collaboration; (4) be based on human subjects; and (5) be conducted in the United States (U.S.). Only studies published between 2014 and 2024 were included to ensure contemporary relevance. Accepted study designs included randomized controlled trials, prospective and retrospective clinical trials, case–control studies, cross-sectional studies, and case series or reports. Reviews, editorials, letters, conference abstracts, animal studies, and other secondary sources were excluded to maintain a focus on original research. Consistent with PRISMA-ScR guidance, we excluded purely educational IPE reports unless they directly examined clinical collaboration involving dental professionals.

As summarized in Table 2, tailored search terms were used across all three databases to identify relevant literature. In PubMed, search terms such as “interprofessional” and “multidisciplinary” were applied to locate studies addressing clinical outcomes and healthcare quality. In SCOPUS and Web of Science, the search incorporated terms like “interprofessional” and “interdisciplinary” with an emphasis on clinical outcomes involving dentists and dental specialists. All searches were restricted to English-language publications.

Two authors (MH and MT) independently screened all retrieved articles using a standardized screening form, identifying 25 from PubMed, 588 from SCOPUS, and 75 from Web of Science. These were then narrowed down to 20, 230, and 67, respectively, after excluding studies published before 2014. A secondary screening was conducted by the same two authors to ensure consistency in applying the inclusion and exclusion criteria. Any disagreements were resolved through discussion, and unresolved discrepancies were adjudicated by a third reviewer. The final article selection was based on relevance to study design, types of healthcare sectors represented, reported challenges and benefits, and the outcomes described. To reduce bias, a third independent reviewer (AM) validated the final article selection and cross-checked extracted data for accuracy and completeness.

A standardized data-charting form was piloted to ensure consistency. Extracted variables included: author(s), year, study design, population, healthcare setting, team composition, outcomes assessed, and reported barriers/facilitators. Data were charted independently by two reviewers (MH and MT) and verified by a third (AM). This rigorous, multi-stage process ensured a transparent, reproducible synthesis of studies addressing the integration of dentists within interprofessional healthcare teams.

## 3. Results

### 3.1. Article Selection

**Figure 1:**
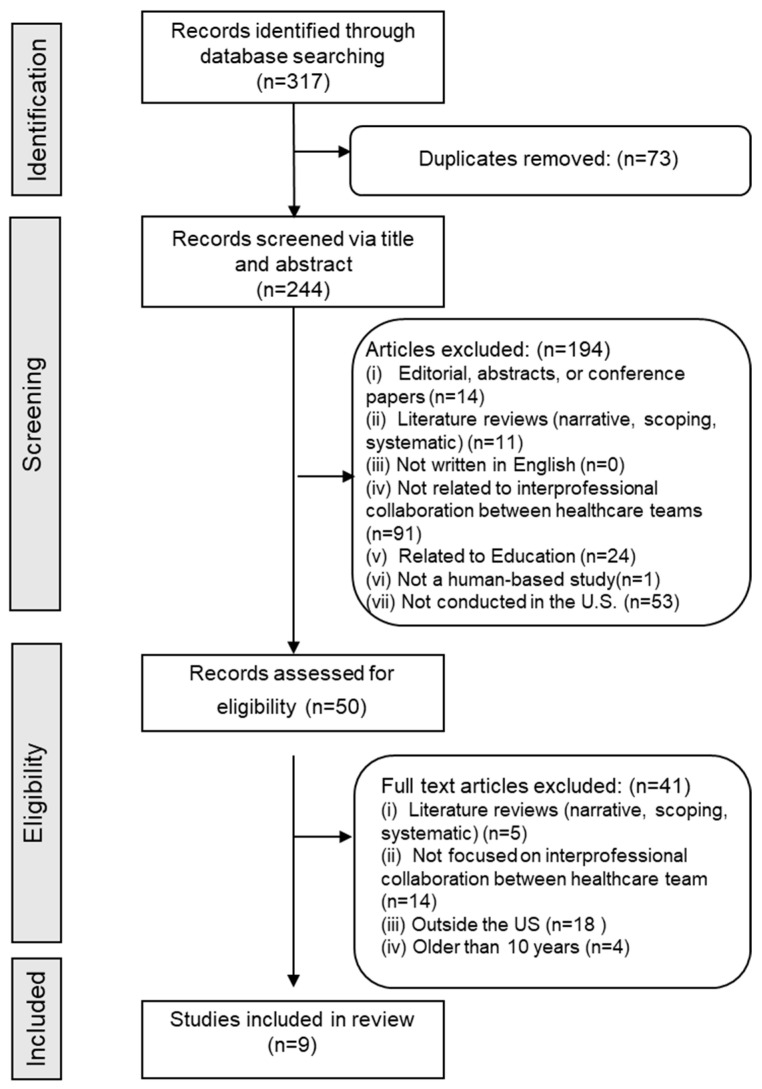
Flowchart of the article selection process.

The PRISMA-ScR flow diagram (Figure 1) summarizes the article selection and screening. A total of 317 titles and abstracts were initially screened. After removing duplicates, 244 unique articles remained. These were reviewed, resulting in the exclusion of 194 articles for the following reasons: 14 were editorials, abstracts, or conference papers; 11 were literature reviews; 91 did not focus on interprofessional collaboration among healthcare teams; 1 was not based on human studies; 53 were conducted outside the U.S.; and 24 were related to education.

Fifty articles were selected for full-text review. After a second round of screening, 41 additional articles were excluded: 5 were literature reviews; 14 did not examine interprofessional collaboration; 18 were conducted outside the U.S.; and 4 were published before 2014. Ultimately, nine studies met all inclusion criteria and were included in the final analysis (Figure 1).

### 3.2. Study Characteristics

Table 3 outlines the various types of IPC models identified across the nine included studies. Examples include partnerships among primary care providers (PCPs), nurses, and dentists, which reflect integrated teamwork within primary care. More complex interdisciplinary models, such as those involving PCPs, physician assistants, dentists, and dental hygienists, demonstrate the multifaceted nature of IPC and highlight ongoing efforts to enhance healthcare delivery through collaboration.

Barriers to IPC (Table 4) were grouped into four domains: system level, organizational, inter-individual, and individual level. The most prevalent obstacles were organizational and inter-individual barriers, particularly the absence of shared EHR systems, limited role clarity, and communication breakdowns among providers. System-level challenges, such as inadequate training and limited policy support, were identified in three studies. Individual-level barriers, including low provider motivation or limited understanding of IPC value, were reported less frequently but remained relevant to implementation success.

Facilitators of IPC are presented in Table 5. Key enablers included the availability of funding and financial resources, supportive institutional and governmental policies, and the implementation of educational programs designed to promote collaboration within healthcare facilities. These factors were particularly influential in supporting models involving dentists, nurses, and primary care providers.

Table 6 further details study characteristics, design, and participant demographics. Case reports accounted for 44% of included studies, ethnographies 22%, and cross-sectional designs 22% (Table 7). Participant ages ranged from 9 to 82 years, reflecting a broad spectrum of patient populations (Table 6). The studied health domains included oral-systemic disease links such as diabetes, sleep apnea, early childhood caries, general oral health integration, and pregnancy-related oral health (Table 7).

### 3.3. Thematic Synthesis of Findings

The analysis of nine studies on IPC in healthcare highlighted multiple successes in integrating dentists into healthcare teams, yielding measurable benefits for patient well-being and care quality (Table 8).

Alexander et al. [43] reported a 98% reduction in apnea–hypopnea index scores and a 75% increase in quality-of-life scores among pediatric sleep apnea patients through interdisciplinary treatment. Similarly, Wood et al. [49] found that shared expertise within federally qualified health center teams improved patient outcomes and satisfaction, while Dolce et al. [41] demonstrated that the nurse practitioner–dentist model enhanced prevention, awareness, and education. These examples underscore the positive impact of collaborative healthcare models on patient health.

Gibson et al. [44] illustrated the clinical value of interdisciplinary collaboration in managing complex conditions such as cleft palate, where coordinated treatment among an orthodontist, periodontist, and general dentist improved both functional and aesthetic outcomes. Inglehart et al. [47] emphasized the importance of integrating oral health into holistic, patient-centered care—particularly during pregnancy—reinforcing the need to incorporate dental health into broader health management strategies. 

Across studies, effective communication, shared learning, and cross-disciplinary education were identified as critical facilitators of safe, coordinated, and comprehensive care. These elements enable all providers to recognize the contribution of oral health to overall well-being and to collaborate more efficiently in addressing systemic health concerns. Despite these successes, persistent barriers continue to limit the full implementation of IPC involving dental professionals. Communication challenges remain prominent: Inglehart et al. [47] identified limited oral-health training among non-dental providers as a barrier to interdisciplinary care during pregnancy, while Horowitz et al. [45] reported insufficient engagement of hygienists and dentists in addressing early childhood caries, underscoring the need for improved communication and professional development. The absence of integrated EHR systems constrains responsiveness among providers and disrupts care continuity, whereas unclear professional roles and the lack of standardized protocols contribute to inconsistent patient management [49].

Knowledge gaps in systemic disease management, such as diabetes, were highlighted by Shimpi et al. [46], who emphasized the importance of integrated care frameworks and enhanced professional education. Referral inefficiencies also pose challenges; Long et al. [48] found that improving referral acceptance requires clearer triage protocols and strengthened interprofessional collaboration. Finally, Griffin et al. [42] and Dolce et al. [41] advocated for systemic interventions, including structured communication pathways and EHR integration, to facilitate seamless information exchange and coordination across care settings. 

## 4. Discussion

This scoping review of nine studies on IPC highlights how integrating dentists into healthcare teams enhances patient outcomes and care coordination. The synthesis confirms the value of dental integration while identifying persistent organizational and policy barriers to widespread adoption. These findings are consistent with broader literature emphasizing that sustainable oral health integration must address both access disparities and system-level coordination challenges [24].

IPC consistently improved outcomes and continuity of care. Their significance does not just lie in the outcomes themselves but in the mechanisms enabling them—shared accountability, interdisciplinary communication, and patient-centered coordination. The evidence indicates that interprofessional models may help mitigate medical errors and enhance diagnostic accuracy, as clinical decisions are informed by diverse expertise. These findings also align with broader evidence demonstrating the importance of preventive oral health interventions, such as those evaluated by Lile et al. [50], who compared natural mouthrinses and chlorhexidine for plaque management, as part of a comprehensive, team-based approach to improving patient outcomes. This pattern is consistent with work in other domains, where improved team function—grounded in trust, psychological safety, and shared mental models—has been shown to enhance both patient and organizational outcomes across clinical settings [33]. Collectively, these benefits underscore the central role of oral health in whole-person care and support calls for stronger inclusion of dental professionals in primary and specialty healthcare teams.

The main barriers identified are interrelated system-level issues rather than isolated challenges. Fragmented communication, limited IPE, and non-interoperable EHRs persist as primary obstacles. Financial and policy misalignments, such as separate reimbursement structures, further restrict integration. These factors compound each other: inadequate training weakens understanding of oral–systemic connections, while data silos and unclear roles disrupt care continuity and efficiency. Overall, the issue is not conceptual support for IPC but the lack of enabling systems to implement it. Reforms in education, health information infrastructure, and policy alignment are essential to translate the demonstrated benefits of IPC into consistent, sustainable practice.

### 4.1. Implications

The findings of this review carry several important implications for healthcare practice, education, and policy. First, the demonstrated benefits of IPC affirm the need to embed oral health within broader medical and public health agendas. Health systems and educational institutions should promote shared training opportunities and team-based practice models that normalize collaboration among dentists, physicians, nurses, pharmacists, and other providers.

Second, addressing the identified barriers requires multi-level strategies. At the institutional level, implementation of interoperable EHR systems is critical for enabling seamless data exchange and coordinated treatment planning. At the educational level, both medical and dental curricula should integrate interprofessional competencies and case-based training to foster communication and mutual understanding early in professional development. At the policy level, regulatory bodies and payers should incentivize interprofessional practice through shared reimbursement mechanisms, integrated quality metrics, and funding for team-based care pilots. Such approaches would help translate the conceptual value of IPC into operational practice.

Finally, research and evaluation frameworks should shift from descriptive case reporting to robust, mixed-method studies that quantify clinical and economic outcomes of interprofessional dental integration. This will strengthen the evidence base for policy and inform scalable implementation models.

### 4.2. IPE and Training Models

IPE is central to fostering the collaborative competencies necessary for effective integration of dental professionals within healthcare teams. The World Health Organization’s Framework for Action on Interprofessional Education and Collaborative Practice (2010) [1] defines IPE as occasions when “students from two or more professions learn about, from, and with each other” to enable effective collaboration and improve health outcomes. This approach encourages the development of mutual understanding, respect, and communication across disciplines—skills that are essential for bridging the divide between oral and general healthcare.

Several U.S.-based models have demonstrated success in promoting oral–systemic collaboration. The Smiles for Life oral health curriculum, endorsed by the Society of Teachers of Family Medicine, equips non-dental professionals with foundational knowledge of oral–systemic health and preventive oral care [51]. Similarly, studies by Reeves et al. [52] and Haber et al. [53] highlight the value of simulation-based and case-driven IPE interventions that improve teamwork, clinical communication, and shared accountability. These educational efforts align with calls from national organizations to strengthen interprofessional dental education and community-based learning as strategies for addressing access inequities and preparing a future workforce capable of collaborative care [24].

To strengthen IPC, educational institutions should incorporate oral health content into medical, nursing, and pharmacy curricula and expand team-based learning opportunities. Simulation exercises, joint clinical rotations, and problem-based learning modules can help future providers develop a shared understanding of oral–systemic interdependence. Embedding IPE across disciplines ensures that oral health is viewed as an integral component of comprehensive, patient-centered care.

### 4.3. Strengths and Limitations

This scoping review has several strengths. It systematically identified and analyzed primary studies spanning multiple care settings and populations, providing a comprehensive overview of the current interprofessional practices involving dentists. The inclusion of diverse study designs, ranging from case reports to observational studies, allowed for triangulation of findings across contexts. The use of rigorous, pre-defined inclusion and exclusion criteria, guided by the PRISMA-ScR framework, further enhances transparency and reproducibility.

However, limitations should be acknowledged. First, the review included studies published up to September 2024; therefore, more recent publications on this evolving topic may not have been captured. This reflects a standard practice in review methodology, where a defined cutoff ensures methodological consistency despite ongoing publication of new studies. The review was also limited to studies conducted within the U.S. and published in English, which may limit the generalizability of the findings to global contexts. This focus reflects the intention to analyze systems governed by similar reimbursement and regulatory structures but precludes insights from countries with more integrated health models (e.g., the U.K., Canada, or Australia). The findings primarily reflect systems shaped by U.S.-specific policy, reimbursement, and EHR infrastructures. Consequently, the generalizability of these results to countries with different healthcare financing or workforce models may be limited. However, several facilitators, such as role clarification, shared information systems, and interprofessional education, are broadly applicable across health systems. Additionally, the relatively small number of eligible studies and the predominance of descriptive designs constrain the ability to draw firm causal inferences about the impact of dentist integration. Few studies provided quantitative outcome measures or rigorous quality assessments, underscoring the need for future research employing controlled or longitudinal designs. Furthermore, while this review primarily examined provider-level collaboration, future research should incorporate patient perspectives to capture how interprofessional care is experienced at the individual level. Exploring patients’ perceptions of communication, continuity of care, satisfaction, and trust in collaborative healthcare environments would offer critical insights into the real-world impact of integrated dental–medical models. Qualitative and mixed-methods studies examining these experiences could inform strategies that enhance both patient engagement and the delivery of person-centered care.

## 5. Conclusions

This scoping review demonstrates that integrating dentists within interprofessional healthcare teams enhances diagnostic accuracy, preventive care, and patient satisfaction. However, current evidence base remains limited, with few large-scale or longitudinal studies evaluating the long-term impact of such collaboration.

Future work should move beyond descriptive designs to identify effective, scalable implementation strategies. Priorities include developing shared electronic health records, aligning reimbursement models, and embedding interprofessional education across health disciplines. By clarifying dentistry’s role within team-based care, future initiatives can strengthen oral–systemic integration and advance oral health as a core element of comprehensive healthcare.

## Figures and Tables

**Table 1:** Inclusion/exclusion criteria for article selection.

Criteria	Inclusion	Exclusion
Research Focus	Peer-reviewed articles Focused on the outcomes, benefits, and challenges encountered by an interprofessional healthcare team that involves dentists Human subject research Studies conducted in the United States	Full text not available Abstract not available Focused on interprofessional education Non-dental and non-medical professions
Study Design	Randomized controlled clinical trials Prospective clinical trials Retrospective clinical trials Case–control observational studies Cross-sectional studies Experimental Case series Case reports	Narrative reviews Literature reviews Scoping reviews Meta-analyses Systematic reviews Editors’ choices Editorial comments Opinions Letters to the editor Replies to the author/editor Book abstracts Conference abstracts Ongoing studies Animal models
Language	English language	Non-English language
Publication Year	Published between 2014 and 2024	Published before 2014

**Table 2:** Summaries of the search strategies utilized for the three databases used in this study.

Databases (Coverage Dates)	Search Strategies	Number of Articles Found
PubMed (January 2014–September 2024)	(“interdisciplinary” [Title/Abstract] OR “interprofessional” [Title/Abstract] OR “multiprofessional” [Title/Abstract] OR “multi-professional” [Title/Abstract] OR “inter-professional” [Title/Abstract]) AND (“healthcare” [Title/Abstract] OR “clinical outcomes” [Title/Abstract] OR “quality outcomes” [Title/Abstract] OR “quality of healthcare” [Title/Abstract] OR “patient care” [Title/Abstract] OR “healthcare quality improvement” [Title/Abstract]) AND (Dent * [tiab]) AND ((“Dentists” [Mesh]) OR (“Orthodontists” [Mesh]))	20
SCOPUS (January 2014–September 2024)	TITLE-ABS-KEY ((“interdisciplinary” OR “interprofessional” OR “multiprofessional” OR “multi-professional” OR “inter-professional”) AND (“healthcare” OR “clinical outcomes” OR “quality outcomes” OR “quality of healthcare” OR “patient care” OR “healthcare quality improvement”) AND (“Dent *”) AND (“Dentists” OR “Dentist” OR “Prosthodontists” OR “Dentists, Prosthetic” OR “Dentist, Prosthetic” OR “Prosthetic Dentist” OR “Prosthetic Dentists” OR “Dentists, Restorative” OR “Dentist, Restorative” OR “Restorative Dentist” OR “Restorative Dentists” OR “Dentists, Pediatric” OR “Dentist, Pediatric” OR “Pediatric Dentist” OR “Pediatric Dentists” OR “Periodontists” OR “Periodontist” OR “Orthodontist” OR “Orthodontists” OR “Dentofacial Orthopedists” OR “Dentofacial Orthopedist” OR “Orthopedist, Dentofacial” OR “Orthopedists, dentofacial”))	230
Web of Science (January 2014–September 2024)	(TS = (“interdisciplinary” OR “interprofessional” OR “multiprofessional” OR “multi-professional” OR “inter-professional”)) AND (TS = (“healthcare” OR “clinical outcomes” OR “quality outcomes” OR “quality of healthcare” OR “patient care” OR “healthcare quality improvement”)) AND (TS = (Dent *)) AND (TS = (“Dentists” OR “Dentist” OR “Prosthodontists” OR “Dentists, Prosthetic” OR “Dentist, Prosthetic” OR “Prosthetic Dentist” OR “Prosthetic Dentists” OR “Dentists, Restorative” OR “Dentist, Restorative” OR “Restorative Dentist” OR “Restorative Dentists” OR “Dentists, Pediatric” OR “Dentist, Pediatric” OR “Pediatric Dentist” OR “Pediatric Dentists” OR “Periodontists” OR “Periodontist” OR “Orthodontist” OR “Orthodontists” OR “Dentofacial Orthopedists” OR “Dentofacial Orthopedist” OR “Orthopedist, Dentofacial” OR “Orthopedists, dentofacial”))	67

**Table 3:** Different types of interprofessional collaboration identified.

Types of Interprofessional Collaboration	Number of Reviews	Author (Year)
PCP *–nurse–dentist	1	Dolce et al., 2020 [41]
PCP–pharmacist–dentist	1	Griffin et al., 2022 [42]
PCP–medical specialist–dentist	1	Alexander et al., 2019 [43]
Dentist–dental specialist	1	Gibson et al., 2015 [44]
Dentist–hygienist	3	Horowitz et al., 2017 [45] Shimpi et al., 2021 [46] Inglehart et al., 2022 [47]
PCP–dentist–hygienist	1	Inglehart et al., 2022 [47]
PCP–dentist	1	Long et al., 2014 [48]
PCP–physician assistant–dentist–hygienist	1	Wood et al., 2020 [49]

* PCP: primary care provider.

**Table 4:** Barriers to interprofessional collaboration.

Barrier Category	Common Barriers	Number of Studies Reporting
System level	Financial constraints, lack of political support, inadequate training	3
Organizational	Lack of time/resources, no shared EHR systems, missing protocols	6
Inter-individual	Poor communication, unclear roles, lack of team cohesion	6
Individual level	Low follow-up, lack of motivation, limited understanding of interprofessional value	2

**Table 5:** Facilitators of interprofessional collaboration.

Facilitator Category	Facilitator	Description
System level	Supportive policies & funding	Policies and budgets enabling collaborative practices
	Educational infrastructure	Curriculum or continuing education supporting interprofessional care
Organizational	Integrated EHR systems	Tools that promote data sharing and continuity of care
	Team-based care structures	Redesigned workflows to include collaborative checkpoints
Inter-individual	Shared goals and mutual respect	Collaborative decision-making based on shared patient outcomes
	Role clarity	Clear understanding of responsibilities within care teams
Individual level	Positive provider attitude	Willingness to collaborate and appreciation of others’ expertise

**Table 6:** Summary of basic characteristics of the studies.

Author (Publication Year)	Sample Size	Age Range (Year)	Study Aims
Alexander et al., 2019 [43]	2	9	Examine the interdisciplinary approach required to diagnose and treat severe pediatric obstructive sleep apnea
Dolce et al., 2020 [41]	31	65–82	Observe if the NPD model implementation led to improving patient health outcomes
Gibson et al., 2015 [44]	1	26	Observe the effect of interdisciplinary healthcare team on the desired result of patient’s treatment
Griffin et al., 2022 [42]	1	64	Discuss the importance of collaborating between patient’s medical and dental needs
Horowitz et al., 2017 [45]	37	-	Gain understanding of hygienists’ and dentists’ perspectives about children’s oral health
Inglehart et al., 2022 [47]	1	-	Observe how socioeconomic changes in the U.S. contribute to dentistry and oral health disparities
Long et al., 2014 [48]	1000	-	Determine general dentists’ attitudes towards AAP oral health guidelines
Shimpi et al., 2021 [46]	854	18–80	Integrate care delivery with diabetes or prediabetes that can impact oral conditions
Wood et al., 2020 [49]	8	31–58	Investigate the perceptions of interprofessional healthcare providers on how oral healthcare was integrated

**Table 7:** Distribution of studies by location, health condition, and study design.

Author (Year)	Country (State)	Health Condition/Focus	Study Design
Alexander et al., 2019 [43]	U.S. (South Carolina)	Pediatric sleep apnea	Case Report
Dolce et al., 2020 [41]	U.S. (Boston)	Hypertension and/or type 2 diabetes	Quasi-experimental
Gibson et al., 2015 [44]	U.S. (Boston)	Cleft palate/esthetic rehabilitation	Case report
Griffin et al., 2022 [42]	U.S. (Salt Lake City)	Medication-related osteonecrosis of the jaw (MRONJ)	Case Report
Horowitz et al., 2017 [45]	U.S. (Maryland)	Early childhood caries	Ethnography
Inglehart et al., 2022 [47]	U.S. (Michigan)	Oral health disparities and pregnancy	Case Report
Long et al., 2014 [48]	U.S. (North Carolina)	Pediatric oral health guidelines	Cross Sectional
Shimpi et al., 2021 [46]	U.S. (Wisconsin)	Diabetes–oral health link	Cross Sectional
Wood et al., 2020 [49]	U.S. (Iowa)	Integrated oral care models	Ethnography

**Table 8:** Overview of studies examining interprofessional collaboration involving dental professionals.

Author (Year)	Study Participant	Benefits/Positive Effects	Challenges/Barriers	Strategies to Overcome Challenges	Dentist’s Challenges	Outcomes
Alexander et al., 2019 [43]	Pediatricians, Otolaryngologists, Sleep Physicians, Dentists, Speech Pathologists	Improved quality of life, enhanced cognitive function, better sleep study outcomes.	Initially dismissed symptoms; limited access to diagnostic tools; coordination and communication difficulties; treatment compliance; need for long-term follow-up.	Interdisciplinary collaboration leveraging diverse expertise; comprehensive sleep medicine training to improve medical diagnostic awareness; patient advocacy.	Recognition of Obstructive Sleep Apnea (OSA) symptoms; coordination with medical professionals; multidisciplinary treatment planning; patient education and compliance.	Demonstrated that interdisciplinary treatment involving dentists, otolaryngologists, and myofunctional therapists significantly improved sleep study findings and quality of life in pediatric OSA patients.
Dolce et al., 2020 [41]	Nurses and Dentists	Person-centered care model; “one stop” integrated health delivery.	Lack of integrated electronic health records between medical and dental providers.	Development of a shared electronic medical and dental record system	Limited preparedness among dentists for medical billing procedures.	The Nurse Practitioner–Dentist (NPD) model served as an early prototype for promoting interprofessional healthcare delivery.
Gibson et al., 2015 [44]	Orthodontics, Surgeons, Dentists	Optimized treatment outcomes through interdisciplinary collaboration.	Complex treatment objectives; potential for patient overwhelm.	Multi-provider treatment planning with shared visualization tools.	Managing esthetic challenges; addressing nocturnal bruxism.	Integration of Surgically Facilitated Orthodontic Therapy (SFOT) provided outcomes comparable to surgical interventions without surgery.
Griffin et al., 2022 [42]	Pharmacists, Physicians, Dentists	Reduced provider-patient conflict; improved communication.	Inconsistent communication among providers; lack of standardized protocols.	Enhanced communication pathways between pharmacists and dentists to avoid conflicting advice on Medication-Related Osteonecrosis of the Jaw (MRONJ).	Ensuring follow-through in patient management	Highlighted the importance of coordinated communication between dentists, pharmacists, and physicians regarding MRONJ.
Horowitz et al., 2017 [45]	Hygienists and Dentists	Addressing nationwide early childhood caries (ECC).	Low oral health literacy among parents; limited professional participation	Targeted communication strategies; early intervention programs; dentist training.	Professional disagreement on preventive topics (e.g., fluoridation).	Found traditional approaches insufficient for ECC prevention, emphasizing the need for broader collaborative efforts.
Inglehart et al., 2022 [47]	Dentists, Hygienists,. Medical professionals	Growing collaboration between dental, medical, and mental health providers.	Discomfort among non-dental providers in discussing oral health.	Improvement of shared electronic health record systems.	Limited interprofessional relationships (only 20% of dentists reported collaboration with OB-GYNs).	Reinforced recognition that oral health is directly related to mental and physical health.
Long et al., 2014 [48]	Pediatricians and Dentists	Improved coordination for children’s oral health.	Limited knowledge of American Academy of Pediatrics (AAP) oral health guidelines among dentists.	Increased exposure to infants during dental training.	Reluctance among dentists to accept medical referrals.	General dentists expressed positive views about pediatrician involvement in promoting children’s oral health.
Shimpi et al., 2021 [46]	Hygienists and Dentists	Increased understanding of oral-systemic links, especially diabetes.	High prevalence of dental-diabetes comorbidities; limited systemic screening.	Development of national frameworks and educational initiatives for integrated care	Lack of diabetes screening in dental practices despite regular hypertension screening.	Dental professionals demonstrated positive attitudes towards medical-dental integration.
Wood et al., 2020 [49]	Federally Qualified Health Center (FQHC) teams: Physicians, Physician Assistants; Dentists; Dental Hygienists	Improved patient outcomes; enhanced collaboration and shared expertise.	Role clarity not well-defined (though not a major barrier).	Provider confirmation of diagnosis through interdisciplinary coordination.	-	Provider perspectives indicated significant benefits of integrating oral healthcare professionals within FQHC care teams.

## Data Availability

No new data were created or analyzed in this study.
